# Influence of Density-Based Topology Optimization Parameters on the Design of Periodic Cellular Materials

**DOI:** 10.3390/ma12223736

**Published:** 2019-11-13

**Authors:** Hugo A. Alvarez, Habib R. Zambrano, Olavo M. Silva

**Affiliations:** 1 Universidad del Norte, Km.5 Vía Puerto Colombia, Barranquilla 0810, Colombia; hzambrano@uninorte.edu.co; 2 Departamento de Engenharia Mecânica, Universidade Federal de Santa Catarina, Florianópolis 88040-900, Brazil; olavo@lva.ufsc.br

**Keywords:** cellular materials, topology optimization, optimization parameters

## Abstract

The density based topology optimization procedure represented by the SIMP (Solid isotropic material with penalization) method is the most common technique to solve material distribution optimization problems. It depends on several parameters for the solution, which in general are defined arbitrarily or based on the literature. In this work the influence of the optimization parameters applied to the design of periodic cellular materials were studied. Different filtering schemes, penalization factors, initial guesses, mesh sizes, and optimization solvers were tested. In the obtained results, it was observed that using the Method of Moving Asymptotes (MMA) can be achieved feasible convergent solutions for a large amount of parameters combinations, in comparison, to the global convergent method of moving asymptotes (GCMMA) and optimality criteria. The cases of studies showed that the most robust filtering schemes were the sensitivity average and Helmholtz partial differential equation based filter, compared to the Heaviside projection. The choice of the initial guess demonstrated to be a determining factor in the final topologies obtained.

## 1. Introduction

Periodic cellular materials (PCM) can be found in nature structures like bones, honeycombs, foams, they are also artificially produced by means of different methods for several applications such as: sandwich plates cores [1] and foams for packaging materials [2,3]. Using PCM, lighter structures are possible to design [4], and new materials with tuned mechanical properties can be created such as bulk or shear modulus maximized, negative Poisson ration and materials with an specified stiffness tensor [5,6].

Cellular materials are increasingly used in many industrial applications. In order to predict the structural behavior in the macroscopic scale, it is necessary to consider a full-scale model taking into account the internal architecture of the cellular material. If the cellular material architecture is implemented into the whole model geometry, then the computational cost for the model become costly. On the other hand, the cellular material structure effect can be taken into account in the material constitutive model using the properties obtained from the homogenization procedure of a single unit cell. To obtain the properties of PCM, it is necessary to implement some mathematical approach, in order to compute the overall material properties, these techniques are called homogenization procedures. Sigmund [5] used the homogenization method to obtain the equivalent mechanical properties for a unit cell, which represent a PCM, this approach defines the material properties in a coarse or macroscopic scale from the properties of its mesostructure or unit cell. The homogenization procedure proposed by Hassani and Hinton [7], considers the equations for obtaining the stiffness tensor by means of an asymptotic expansion, these homogenization equations are expressed as a function of the strain fields and the base material properties.

The topology optimization method was first used for periodic materials design by Sigmund [5]. The first mesostructure obtained by means of this method for continuum-like materials were based on thickness variation of the elements, instead of “artificial density” as in the latest topology optimization procedures [8].

Another popular topology optimization method is the Bidirectional Evolutionary Structural Optimization (BESO). Huang et al. [9] has implemented successfully the method BESO to obtain mesostructures that maximizes bulk and shear modulus. Additionally, the Level Set Method (LSM) was successfully used by Wang et al. [10] to propose a new design procedure for multi-phase materials with prescribed mechanical properties. Although the LSM method does not require the implementation of filters to solve the checkerboard problem, it is common to find some numerical difficulties related to the use of finite differences schemes to solve the evolution problem, for some advanced problems could be complicated to satisfy the Courant-Friedrichs-Lewi condition (CFL). Some authors have implemented the LSM method to solve the minimum compliant design problem (MCD) [11], and practical design applications such as optical and carpet cloaks [12,13].

The most popular technique for structural topology optimization is the density based approach viz. “Solid Isotropic Material with Penalization” (SIMP) developed by Bendsøe, M. P., Sigmund [8]. SIMP method is widely used to solve optimization problems, Zuo and Saitou [14] used this method to solve multi-material MCD problems and Bai and Zuo [15] developed a formulation to analyze hollow structures. Regarding PCM Xia and Breitkopf [16] proposed a Matlab code to obtain material mesostructures maximizing shear and bulk modulus. In the same work, an objective function to generate mesostructures with negative Poisson ratio was also implemented. The code developed by Xia and Breitkopf [16] was based on a Matlab code created by Andreassen et al. [17] for the solution of the minimum compliance design problem. Some other authors have used the same method to design architected cellular materials [6,18,19]. A complete review addressing the topic of topology optimization for architected materials can be found in [20,21].

Ingrassia et al. [22], Chiu et al. [23] studied the influence of optimization parameters in BESO for MCD problems. In the same way, Zuo et al. [24] assessed the performance of MMA (Method of Moving Asymptotes) and GCMMA (Global Convergent Method of Moving Asymptotes) optimization solvers to deal with MCD problems using the SIMP method. In the same work Zuo et al. [24] proposed a hybrid formulation of MMA and GCMMA to improve the efficiency and convergence of the solution. Additionally, several benchmark problems were solved using the SIMP method in references [25,26].

In the present work, we show the dependence of the solutions obtained using the SIMP method on some parameters such as: penalization factor, filtering method to obtain “0–1” (black and white solutions), optimization solvers, mesh size and initial conditions. Additionally, some issues related to the uniqueness and “monotonicity” of the objective function used in the inverse homogenization procedure for PCM were analyzed. Finally, the characteristics of the objective function and the influence of the aforementioned parameters, for inverse homogenization in plane stress problems were studied in this article.

## 2. Homogenization of Periodic Cellular Materials

To obtain the macroscopic mechanical properties of PCM, it is necessary to implement a homogenization procedure in order to estimate the effective stiffness or elasticity tensor in each iteration during the optimization process. If a representative unit cell with periodic boundary conditions is considered, the homogenized elasticity tensor in the domain Ω which represents the material macroscopic properties can be calculated using the asymptotic expansion procedure as follow [9]:

(1) $$C_{ijkl}^{H}=\frac{1}{\left|Y\right|}\int_{\Omega }C_{ijpq}\left({\bar{\varepsilon }}_{pq}^{kl}-{\tilde{\varepsilon }}_{pq}^{kl}\right)d\Omega$$

where, |Y| denotes the cell volume in the domain Ω. ${\bar{\varepsilon }}_{pq}^{kl}$ are the linearly independent unit strains: ${\bar{\varepsilon }}_{pq}^{11}={\left(1\;0\;0\right)}^{T}$,${\bar{\varepsilon }}_{pq}^{22}={\left(0\;1\;0\right)}^{T}$ and ${\bar{\varepsilon }}_{pq}^{12}={\left(0\;0\;1\right)}^{T}$, ${\tilde{\varepsilon }}_{pq}^{kl}$ is the induced strain field and $C_{ijpq}$ is the elasticity tensor for the base material. If an energy based approach is implemented, then, the unit test strains $\varepsilon_{pq}^{A(kl)}$ should be induced on the boundaries which corresponds to the strain fields $\left({\bar{\varepsilon }}_{pq}^{kl}-{\tilde{\varepsilon }}_{pq}^{kl}\right)$. Equation (1) can be re-written in terms of element mutual energies as follow [5,16]:

(2) $$C_{ijkl}^{H}=\frac{1}{\left|Y\right|}\int_{\Omega }C_{pqrs}\varepsilon_{pq}^{A(ij)}\varepsilon_{rs}^{A(kl)}d\Omega$$

Equation (2) can be written on its finite element form, considering a unit cell discretized into *N* finite elements and the displacement solutions $u_{e}^{A(kl)}$ corresponding to the strain fields ${\bar{\varepsilon }}_{pq}^{kl}$ as follow:

(3) $$C_{ijkl}^{H}=\frac{1}{\left|Y\right|}\sum_{e=1}^{N}{\left(u_{e}^{A(ij)}\right)}^{T}k_{e}u_{e}^{A(kl)}$$

where, $k_{e}$ is the element stiffness matrix.

### Elastic Problem Solution

**Figure 1:**
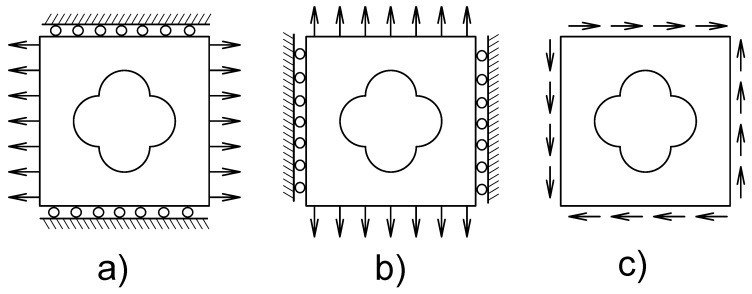
Load cases for the homogenization procedure for 2D problems. (**a**) $\varepsilon^{\left(11\right)}\;=\;\left(1\;0\;0\right)$, (**b**) $\varepsilon^{\left(22\right)}=\left(0\;1\;0\right)$, (**c**) $\varepsilon^{\left(12\right)}=\left(0\;0\;1\right)$.

The strain field ${\tilde{\varepsilon }}_{pq}^{kl}$ is computed from a set of three load cases (for plane stress), as showed in Figure 1, and the equations can be written as follow [18]:

(4) $$\begin{array}{cccc} \; & \nabla \cdot \sigma (u_{ab})=0\;\;\; & \mathrm{on}\;\; & \Omega \\ \; & \sigma \left(u_{ab}\right)\cdot n=\frac{1}{2}\left(e_{a}e_{b}^{T}+e_{b}e_{a}^{T}\right)\cdot n\;\;\; & \mathrm{on}\;\; & \partial \Omega \end{array}$$

where, $u_{ab}$ is the displacements vector, $e_{a}$ is the unit vector along the a−th direction, $\frac{1}{2}\left(e_{a}e_{b}^{T}+e_{b}e_{a}^{T}\right)$ are the tractions on the boundary ∂Ω, and n is a unit vector normal to the boundary.

**Figure 2:**
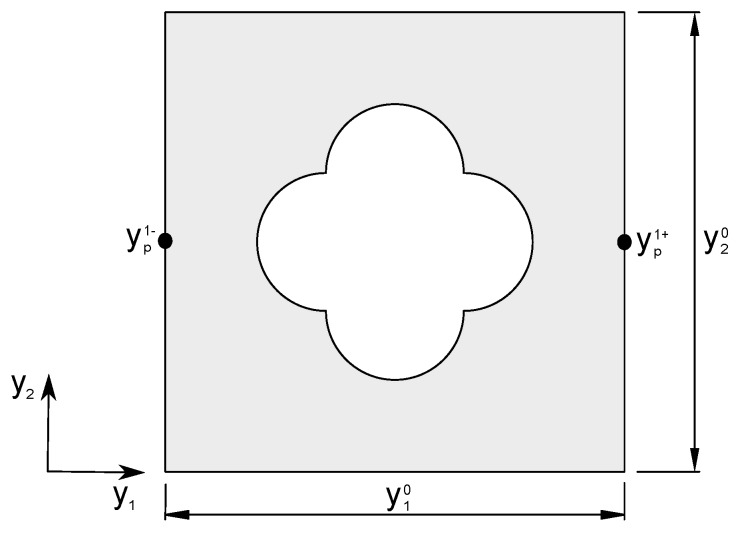
Periodic cell.

The plane stress problem described in Equation (4) is solved using periodic boundary conditions. If it is considered a periodic displacement field, then, the induced strain ${\bar{\varepsilon }}_{pq}^{ij}$ is expressed as the sum of a macroscopic displacement field and a periodic field ${\tilde{u}}_{p}$ [16], as follow:

(5) $$u_{p}={\bar{\varepsilon }}_{pq}^{ij}y_{q}+{\tilde{u}}_{p}$$

where, $y_{q}$ is the length of the cell in the direction *q*. Considering the cell depicted in Figure 2, the displacements on the opposite boundaries are defined by:

(6) $$\begin{array}{c} u_{p}^{k+}={\bar{\varepsilon }}_{pq}^{ij}y_{q}^{k+}+{\tilde{u}}_{p}\\ u_{p}^{k-}={\bar{\varepsilon }}_{pq}^{ij}y_{q}^{k-}+{\tilde{u}}_{p} \end{array}$$

where, superscripts “k+” and “k−” define two pair of opposite parallel boundary surfaces. Eliminating ${\tilde{u}}_{p}$ from Equation (6) the following periodic boundary condition was obtained:

(7) $$u_{p}^{k+}-u_{p}^{k-}={\bar{\varepsilon }}_{pq}^{ij}\Delta y_{q}^{k}$$

this condition relates the displacements for opposite faces of the cell, giving the periodicity condition necessary to model the PCM response. The stiffness tensor is obtained by means of solving Equation (4) with the boundary conditions in Equation (7) and using the homogenization procedure in Equation (1).

## 3. Optimization Model

The SIMP method for topology optimization was implemented. The domain Ω was discretized into *N* finite elements and the design variables were represented by the “artificial density” of each element $\rho_{e}$. Using SIMP, the Young modulus for an element $E_{e}$ is defined as a polynomial function [8,16]:

(8) $$E_{e}\left(\rho_{e}\right)=E_{min}+\rho_{e}^{p}\left(E_{0}-E_{min}\right)$$

where, $E_{0}$ is the Young’s modulus for the base material, $E_{min}$ is a factor to avoid the singularity of the element’s stiffness matrix, and *p* is a penalization factor, $\rho_{e}$ is a continuous scalar value defined in the interval $0\leq \rho_{e}\leq 1$.

The inverse homogenization procedure to obtain PCM can be stated as the following optimization problem [16]:

(9) $$\begin{array}{cccc} \; & \min_{\rho }\; & \; & c(C_{ijkl}^{H}\left(\rho \right))\\ \; & s.t\; & \; & {\mathrm{KU}}^{A(kl)}=F^{\left(kl\right)},k,l=1,2\\ \; & \; & \; & \sum_{e=1}^{N}\nu_{e}\rho_{e}/\left|Y\right|\leq \vartheta \\ \; & \; & \; & 0\leq \rho_{e}\leq 1,e=1,\cdots ,N \end{array}$$

where, $\nu_{e}$ is the element volume, ϑ is the prescribed volume fraction for the PCM, K is the global stiffness matrix $U^{A\left(kl\right)}$ and $F^{\left(kl\right)}$ are the global displacements and forces vector for each load case defined in Figure 1. The authors Xia and Breitkopf [16], Chen et al. [27] and Neves et al. [6] have proposed different objective functions for Equation (9). For the maximization of shear modulus $c=-(C_{1212})$, bulk modulus $c=-0.25(C_{1111}+C_{1122}+C_{2211}+C_{2222})$ or material with negative Poisson ratio $c=C_{1122}-\beta^{l}\left(C_{1111}+C_{2222}\right)$ where β is a fixed parameter defined in the interval 0<β<1 and *l* is the current iteration during the optimization process. In the general case of Equation (9), the inverse homogenization problem formulated to obtain materials with a prescribed stiffness tensor can be stated as [18]:

(10) $$\begin{array}{cccc} \; & \min_{\rho }\; & \; & \left|\right|C_{ijkl}^{0}-C_{ijkl}^{H}{\left(\rho \right))||}^{2}\\ \; & s.t\; & \; & {\mathrm{KU}}^{A(kl)}=F^{\left(kl\right)},k,l=1,2\\ \; & \; & \; & \sum_{e=1}^{N}\nu_{e}\rho_{e}/\left|Y\right|\leq \vartheta \\ \; & \; & \; & 0\leq \rho_{e}\leq 1,e=1,\cdots ,N \end{array}$$

where, $C_{ijkl}^{0}$ is the prescribed stiffness tensor and $C_{ijkl}^{H}$ is the homogenized tensor obtained in the current iteration.

## 4. Sensitivity Analysis

In order to asses different approaches to solve the optimization problem (9), three gradient-based methods were implemented. The methods considered in this work were: Optimality Criteria [8], Method of Moving Asymptotes [28] and the Global Convergent Method of Moving Asymptotes [29]. For implementing the approaches, it is necessary to calculate the derivatives of the objective function and constraint equations with respect to ρ. The derivative of the stiffness tensor $\partial C_{ijkl}\left(\rho \right)/\partial \rho$ was computed using the adjoint method [30] and is given by:

(11) $$\frac{\partial C_{ijkl}\left(\rho \right)}{\partial \rho }=\frac{1}{\left|Y\right|}p\rho_{e}^{p-1}\left(E_{0}-E_{min}\right){\left(u_{e}^{A(ij)}\right)}^{T}k_{0}u_{e}^{A(ij)}$$

where, $k_{0}$ is the element stiffness matrix for the solid material. The first constraint in Equation (9) is the equilibrium condition and is forced during the finite element solution. If a uniform mesh is implemented with an element volume $v_{e}=1$, thus, the volume derivative $\partial V/\partial \rho_{e}=1$. The derivative of the function $\left|\right|C_{ijkl}^{0}-C_{ijkl}^{H}\left(\rho \right){\left|\right|}^{2}$ can be obtained considering the norm definition of a fourth order tensor as the double inner product ${\left||T|\right|}^{2}=T:T$, as follow:

(12) $$\begin{array}{cc} \; & \frac{\partial }{\partial \rho }\left|\right|C_{ijkl}^{0}-C_{ijkl}^{H}\left(\rho \right){\left|\right|}^{2}=\frac{\partial }{\partial \rho }\left[\left(C_{ijkl}^{0}-C_{ijkl}^{H}\left(\rho \right)\right):\left(C_{ijkl}^{0}-C_{ijkl}^{H}\left(\rho \right)\right)\right]\\ \; & \left(C_{ijkl}^{0}-C_{ijkl}^{H}\left(\rho \right)\right):\left(C_{ijkl}^{0}-C_{ijkl}^{H}\left(\rho \right)\right)={\left(C_{ijkl}^{0}-C_{ijkl}^{H}\left(\rho \right)\right)}^{2}\\ \; & \frac{\partial }{\partial \rho }\left|\right|C_{ijkl}^{0}-C_{ijkl}^{H}\left(\rho \right){\left|\right|}^{2}=-2\left(C_{ijkl}^{0}-C_{ijkl}^{H}\left(\rho \right)\right)\frac{\partial C_{ijkl}^{H}\left(\rho \right)}{\partial \rho } \end{array}$$

## 5. Filtering

Three different filtering strategies to obtain manufacturable patterns were compared. The most common filter approaches were taken into account, viz., Field averaging, Heaviside projection and filter based on the solution of the Helmholtz partial differential equation [31,32,33].

### 5.1. Field Averaging

In the field averaging approach the sensitivities are modified as follow:

(13) $$\frac{\hat{\partial c}}{\partial \rho_{e}}=\frac{1}{\max\left(1\times 10^{-3},\rho_{e}\right)\sum_{i\in N_{e}}H_{ei}}\sum_{i\in N_{e}}H_{ei}\rho_{i}\frac{\partial c}{\partial \rho_{i}}$$

where, $r_{min}$ is the filter radius, $N_{e}$ is the set of elements *i* for which the distance $\Delta \left(e,i\right)\leq r_{min}$ and $H_{ei}=\max\left(0,r_{min}-\Delta \left(e,i\right)\right)$ is a weight factor. In addition, the field averaging method can also be applied to $\rho_{e}$, giving:

(14) $${\hat{\rho }}_{e}=\frac{1}{\sum_{i\in N_{e}}H_{ei}}\sum_{i\in N_{e}}H_{ei}\rho_{i}$$

### 5.2. Heaviside Projection Filter

A modification of Equation (14) was proposed by Guest et al. [34]. In this filter the density ${\tilde{\rho }}_{e}$ is projected to a physical density ${\bar{\rho }}_{e}$, where, ${\bar{\rho }}_{e}=1$ if ${\tilde{\rho }}_{e}>0$ and ${\bar{\rho }}_{e}=0$ if ${\tilde{\rho }}_{e}=0$. The following function is introduced in order to use a gradient-based optimization scheme:

(15) $${\bar{\rho }}_{e}=1-e^{-\beta {\tilde{\rho }}_{e}}+{\tilde{\rho }}_{e}e^{-\beta }$$

where, β is a smoothing parameter. To obtain the sensitivity of Equation (15), the derivative of ${\bar{\rho }}_{e}$ with respect to ${\tilde{\rho }}_{e}$, is computed as:

(16) $$\frac{\partial {\bar{\rho }}_{e}}{\partial {\tilde{\rho }}_{e}}=\beta e^{-\beta {\tilde{\rho }}_{e}}+e^{-\beta }$$

### 5.3. Filter Based on Helmholtz Partial Differential Equation

Lazarov and Sigmund [33] used the Helmholtz PDE solution with homogeneous Neumann boundary conditions to propose a new density filter, as follow:

(17) $$\begin{array}{cc} \; & -\frac{1}{2\sqrt{3}}r_{min}^{2}\nabla^{2}\tilde{\psi }+\tilde{\psi }=\psi \\ \; & \frac{\partial \tilde{\psi }}{\partial n}=0 \end{array}$$

where, ψ is the unfiltered design field and $\tilde{\psi }$ is the filtered field. If a sensitivity filter is applied, then $\psi =\rho \frac{\partial c}{\partial \rho }$ and $\tilde{\psi }=\rho \frac{\tilde{\partial c}}{\partial \rho }$.

## 6. Optimization Solvers

The most common approaches for variable updating in gradient-based topology optimization procedures are: Optimality criteria (OC), Method of moving asymptotes (MMA) and Global convergent method of moving asymptotes (GCMMA) [28,29,35]. A comparison of these methods is given in [26].

### 6.1. Optimality Criteria

In the optimality criteria it is implemented an heuristic updating scheme defined as follow:

(18) $$\rho_{e}^{new}=\left\{\begin{array}{c} \max\left(0,\rho_{e}-m\right)\;\mathrm{if}\;\rho_{e}B_{e}^{\eta }\leq \max\left(0,\rho_{e}-m\right)\\ \min\left(1,\rho_{e}+m\right)\;\mathrm{if}\;\rho_{e}B_{e}^{\eta }\geq \min\left(1,\rho_{e}-m\right)\\ \rho_{e}B_{e}^{\eta }\;\;\;\mathrm{otherwise} \end{array}\right.$$

where, *m* is a positive move limit, η is a damping coefficient, and $B_{e}$ is related to the optimality condition as function of Lagrange multiplier λ:

(19) $$B_{e}=\frac{-\frac{\partial c}{\partial \rho_{e}}}{\lambda \frac{\partial V}{\partial \rho_{e}}}$$

### 6.2. MMA and GCMMA Methods

MMA is based on a special type of convex separable approximation, using a first order Taylor series expansion of the objective and constraint equations. In this method, a subset of optimization problems was solved using asymptotes $L_{j}$ and $U_{j}$ as lower and upper bounds in each sub-problem, the values of the asymptotes are normally changed between the iterations. The approximation functions for each sub-problem *i* are chosen as:

(20) $$\begin{array}{cc} \; & f_{i}^{\left(k\right)}\left(\rho \right)=r_{i}^{\left(k\right)}+\sum_{j=1}^{n}\left(\frac{p_{ij}^{\left(k\right)}}{U_{j}^{\left(k\right)}-\rho_{j}}+\frac{q_{ij}^{\left(k\right)}}{\rho_{j}-L_{j}^{\left(k\right)}}\right)\\ \; & p_{ij}^{\left(k\right)}=\left\{\begin{array}{cc} {\left(U_{j}^{\left(k\right)}-\rho_{i}^{\left(k\right)}\right)}^{2}\partial f_{i}/\partial \rho_{i},\;\; & \mathrm{if}\;\partial f_{i}/\partial \rho_{i}>0\\ 0,\;\; & \mathrm{if}\;\partial f_{i}/\partial \rho_{i}\leq 0 \end{array}\right.\\ \; & q_{ij}^{\left(k\right)}=\left\{\begin{array}{cc} 0,\;\; & \mathrm{if}\;\partial f_{i}/\partial \rho_{i}\geq 0\\ -{\left(\rho_{i}^{\left(k\right)}-U_{j}^{\left(k\right)}\right)}^{2}\partial f_{i}/\partial \rho_{i},\;\; & \mathrm{if}\;\partial f_{i}/\partial \rho_{i}<0 \end{array}\right.\\ \; & r_{i}^{k}=f_{i}\left(\rho^{\left(k\right)}\right)-\sum_{j=1}^{n}\left(\frac{p_{ij}^{\left(k\right)}}{U_{j}^{\left(k\right)}-\rho_{j}^{\left(k\right)}}+\frac{q_{ij}^{\left(k\right)}}{\rho_{j}^{\left(k\right)}-L_{j}^{\left(k\right)}}\right) \end{array}$$

The GCMMA is an extension of MMA in which $p_{ij}^{\left(k\right)}$ and $q_{ij}^{\left(k\right)}$ are not zero simultaneously, and they are function of a non-monotonous parameter. GCMMA includes inner iterations, and the approximations used are more conservative. However, the procedure is slower than MMA. More details of these methods can be found in references [28,29].

## 7. Optimization Parameters

The solution of a topology optimization problem using the SIMP method, depends on variables such as the penalization factor *p*, mesh size, filtering scheme, filter radius $r_{min}$, optimization solver, and the initial guess. The value of *p* defines the polynomial order to approximate the objective function and its derivative. For the minimum compliance design problem, the recommended value of *p* is considered as p≥3. Good results have been reported in the literature using the minimum value p=3 [8]. For the inverse homogenization problem *p* is in general defined by trial and error or using the continuation method [32] until to get a convergent 0–1 solution. p=5 was used by Xia and Breitkopf [16] obtaining good results. Filtering schemes provides mesh independence and manufacturable designs [32]. The effects of different filters were evaluated for minimum compliance designs by Andreassen et al. [17] where the savings in memory usage and computational cost for the Helmholtz PDE based filter are highlighted. The Heaviside projection filter is used to achieve a minimum length scale in the optimized design and to promote 0–1 solutions as proposed in [34]. The effects of parameter $r_{min}$ is well known, and in general a small value of $r_{min}$ will produce topologies with “fine details” and larger values yield to coarser topologies. The solver MMA usually reaches a convergent solution in fewer iterations than optimality criteria, but 0–1 solutions are not guaranteed for inverse homogenization problems, using arbitrary initial guesses. GCMMA is an improvement of MMA introducing a non-monotonous approximation, but still requires a careful selection of the initial guess to obtain an stable, convergent and 0–1 solution. In the general inverse homogenization problem, the objective and constraint functions usually consist of non-convex and implicit form of design variables, therefore it is important to select carefully an appropriate solver for a specific problem [24].

### 7.1. Analytical Bounds

The analytical bounds of the objective function for bulk and shear modulus maximization, were computed to obtain reference values to compare the solutions achieved using the different combinations of optimization parameters. The theoretical achievable bounds for shear and bulk modulus maximization considering quasi-isotropic and quasi-homogeneous multi-phase materials were derived by Hashin and Shtrikman [36] and Andreassen et al. [37]:

(21) $$\begin{array}{cc} \; & K^{H}\leq \frac{4GK\nu }{4G+3K(1-\nu )},\\ \; & G^{H}\leq G+\frac{1-\nu }{\frac{6}{5G}\frac{(K+2G)\nu }{\left(3K+4G\right)}-\frac{1}{G}} \end{array}$$

where, $K^{H}$ and $G^{H}$ are the bounds for maximum bulk and shear modulus respectively, *K* and *G* are the base material bulk and shear modulus respectively, and ν is the volume fraction.

### 7.2. Characterization of the Objective Function

**Figure 3:**
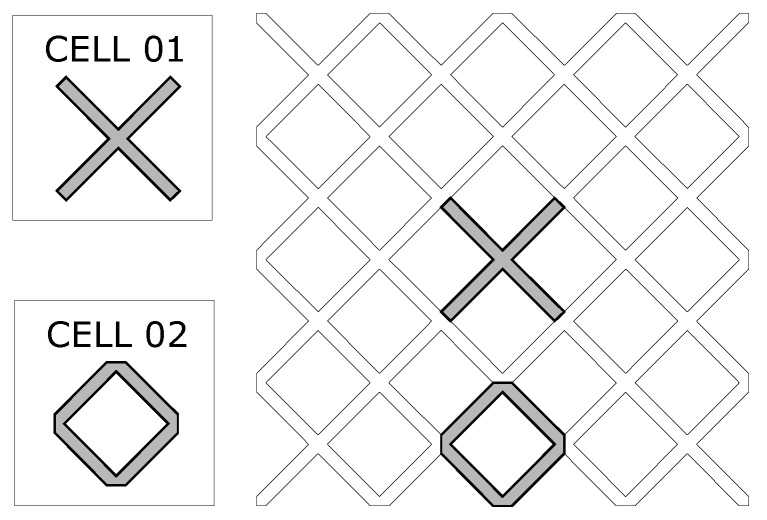
Different ways to select a unit cell in PCM.

Some characteristics of the optimization problem such as: continuity, existence, and uniqueness, have to be considered in order to study the behavior of the optimization parameters. The continuity requirement can be verified looking for the design variables values that make the stiffness matrix K of the Equation (9) not singular. the factor $E_{min}$ in Equation (8) is added in order to avoid the singularity of K. As for the solution uniqueness, it is evident that, the solution is not unique, because there are different unit cells that generate the same PCM (see Figure 3). The appropriate selection of a solver or updating scheme for the design variables, it depends on the characteristics of the objective function. The most relevant is the monotonicity, for instance, a gradient based-method is suitable when the objective function is convex and monotonic. There is not a formal definition of monotonicity for multi-variable functions, but the evaluation of the function behavior modifying the design variables (one at a time) it is accepted as a “monotonicity” test for topology optimization problems [38,39].

**Figure 4:**
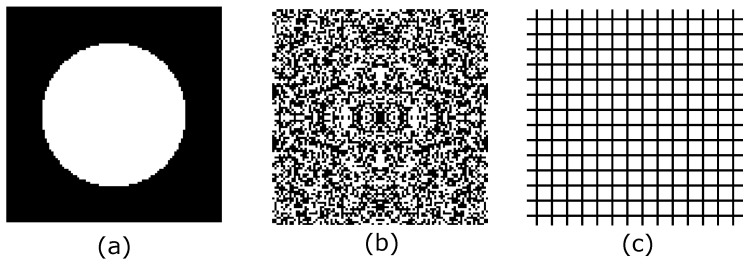
Initial guesses: (**a**) center hole; (**b**) fully random; (**c**) uniform grid.

**Figure 5:**
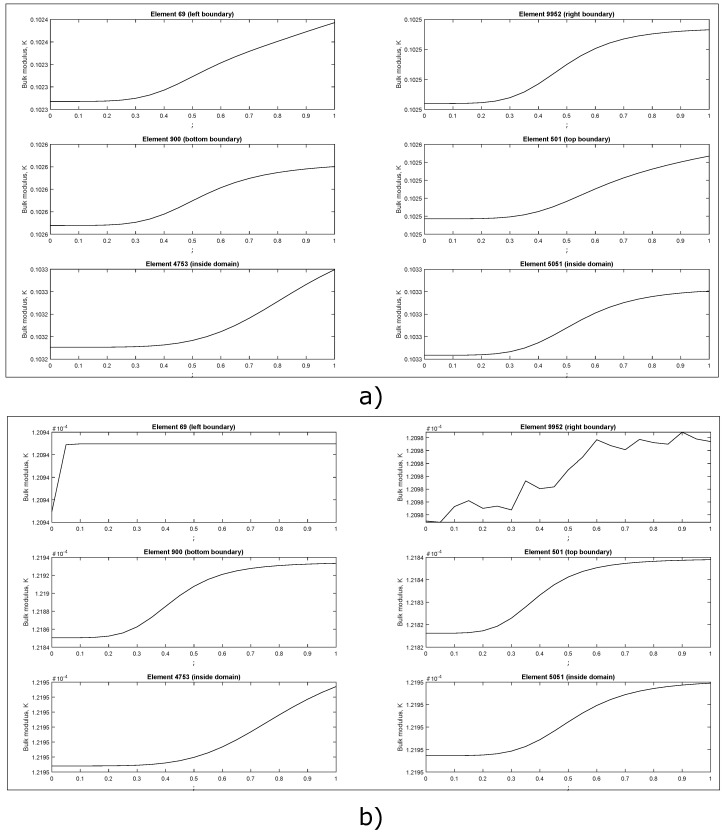
Bulk modulus maximization behavior for 6 representative elements. (**a**) center hole initial guess (**b**) fully random initial guess.

A local “monotonicity” test was performed for the bulk modulus maximization objective function, verifying 100 random elements: 40 at the top, bottom, left and right boundaries and 60 at the center of the domain. $\rho_{e}$ was varied in the interval $0.0\leq \rho_{e}\leq 1.0$, with increments of 0.05, and considering the initial guesses (a) and (b) displayed in Figure 4. In Figure 5 are presented the results. It was observed a monotonic behavior of the objective function when an uniform initial guess (Figure 4a) was considered. If a fully random initial guess (Figure 4b) is used, thus a non-monotonic behavior is obtained for some elements located in the right boundary. The same test was performed for the shear modulus maximization objective function, but it was not obtained any element with non-monotonic behavior.

When the optimization function is “non-monotonic”, thus one or more local optima points exist. To verify the local optima supposition, the objective functions for shear and bulk modulus maximization were evaluated using 100 different random initial guesses similar to Figure 4b, and the obtained topologies were classified into groups by means of the Pearson correlation coefficient $\xi_{c}$ [40], where, all the solutions with $\xi_{c}\geq 0.9$ were considered to be the same topology. All the optimizations were performed using the MMA solver, a mesh size of 100×100 elements, volume fraction ν=0.5, penalization factor p=5.0 and a filter radius $r_{min}=5.0$ using the sensitivity average filter. The Pearson correlation coefficient is defined as:

(22) $$\xi_{c}=\frac{\sum_{m}\sum_{n}\left(A_{mn}-\bar{A}\right)\left(B_{mn}-\bar{B}\right)}{\sqrt{\left(\sum_{m}\sum_{n}{\left(A_{mn}-\bar{A}\right)}^{2}\right)\left(\sum_{m}\sum_{n}{\left(B_{mn}-\bar{B}\right)}^{2}\right)}}$$

where, *A* and *B* are the solutions to be compared, and the subscripts *m* and *n* refers to the element index, $\bar{A}$ and $\bar{B}$ are the mean values of *A* and *B* respectively.

**Figure 6:**
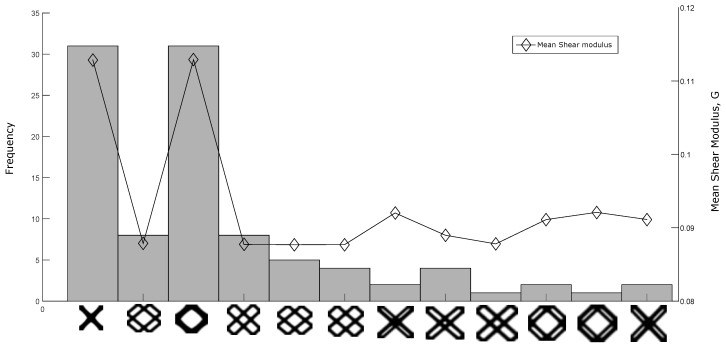
Histogram of 100 runs with the obtained groups for shear modulus maximization. The left axis is the frequency and the right axis is the mean objective function value.

**Figure 7:**
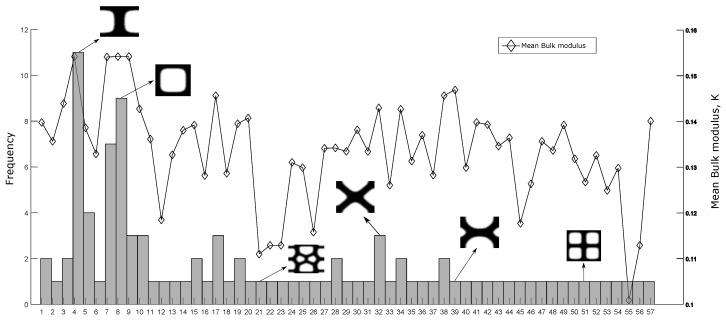
Histogram of 100 runs with the obtained groups for bulk modulus maximization. The left axis is the frequency and the right axis is the mean objective function value. Only 6/57 representative topologies are displayed for clarity.

**Figure 8:**
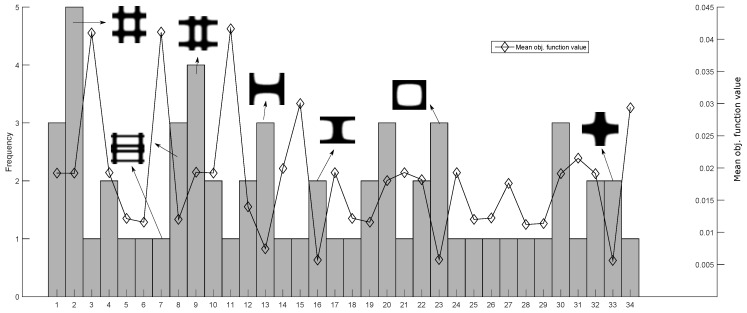
Histogram of 100 runs with the obtained groups for the general inverse homogenization objective function. The left axis is the frequency and the right axis is the mean objective function value. Only 7/34 topologies are displayed for clarity.

The results are presented in Figure 6 and Figure 7, where the existence of multiple local optima points is illustrated in a frequency diagram. The representative topologies for some groups and its corresponding mean objective function value are displayed in the same figures. In Figure 6 and Figure 7 it can be observed that the solutions with the best mean optimum value have a higher probability to be achieved for the shear and bulk modulus maximization, when the optimization was started from a random initial guess. Additionally it was observed that the same periodic material can be defined by different unit cells as defined in Figure 3, confirming the non-uniqueness of the solution. The objective functions for bulk and shear modulus maximization described in Section 3 are particular cases of the objective function for the general inverse homogenization problem stated in Equation (10), which is assumed more complex to solve than its particular cases. In order to compare the objective function for bulk modulus maximization and the function for general inverse homogenization, it was performed the same analysis presented in Figure 6 and Figure 7, using the stiffness tensor $C_{1111}^{0}=C_{2222}^{0}=0.3232,\;C_{1122}^{0}=C_{2211}^{0}=0.0470,\;C_{1212}^{0}=0.0276$, obtained with the bulk modulus maximization objective function and a fully random initial guess. In this analysis, not all the solutions were feasible, only 61/100 runs were 0–1 topologies, according to the following criteria:

(23) $$N/N_{\rho }^{tol}\geq f$$

where, *N* is the total number of elements, $N_{\rho }^{tol}$ is the number or elements with a density value within a tolerance tol and *f* is the required fraction. The results are presented in Figure 8, only feasible solutions were considered, in this test, the global optima it was not the most frequent solution, instead, it was found an scaled version of itself as the most common solution.

**Figure 9:**
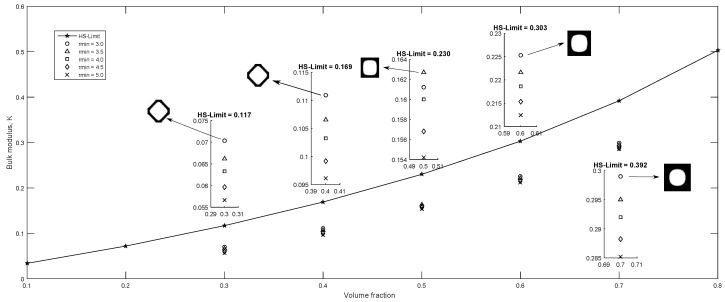
Maximum bulk modulus using the center hole initial guess, 0.3≤ν≤0.7 and 3.0≤rmin≤5.0 and, zoomed sub-plots are displayed for each volume fraction for clarity.

**Figure 10:**
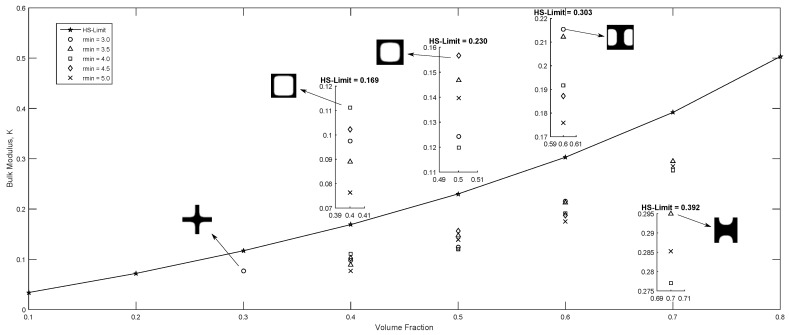
Maximum bulk modulus using the fully random initial guess, 0.3≤ν≤0.7 and 3.0≤rmin≤5.0 and, zoomed sub-plots are displayed for each volume fraction for clarity.

Additionally to the initial guess, $r_{min}$ has an important effect in the solution (magnitude and topology), therefore, an additional study was carried out using five different filter radius in conjunction with five volume ratios, and considering the bulk modulus maximization objective function, for the initial guesses shown in Figure 4a,b, the volume fraction and filter radius were varied within the intervals (0.3≤v≤0.7) and ($0.3\leq r_{min}\leq 5.0$) respectively. The other parameters were defined using the same values implemented to obtain the topologies in Figure 7. The results are presented in Figure 9 for the initial guess in Figure 4a and in Figure 10 for the initial guess in Figure 4b. $r_{min}$ values that did not appear in the Figure 10 are non-convergent solutions. Additionally the Hashin and Shtrikman [36] analytic bounds are presented in Figure 9 and Figure 10. It is observed in Figure 9 that in general the highest bulk modulus was obtained with the minimum filter radius when the iteration process started from a initial guess near to the optimum such as in Figure 4a, this is not valid for the fully random initial guess in Figure 4b, where, the best optima is not necessarily obtained with the lowest $r_{min}$ value as observed in Figure 10.

## 8. Interaction among Parameters

**Figure 11:**
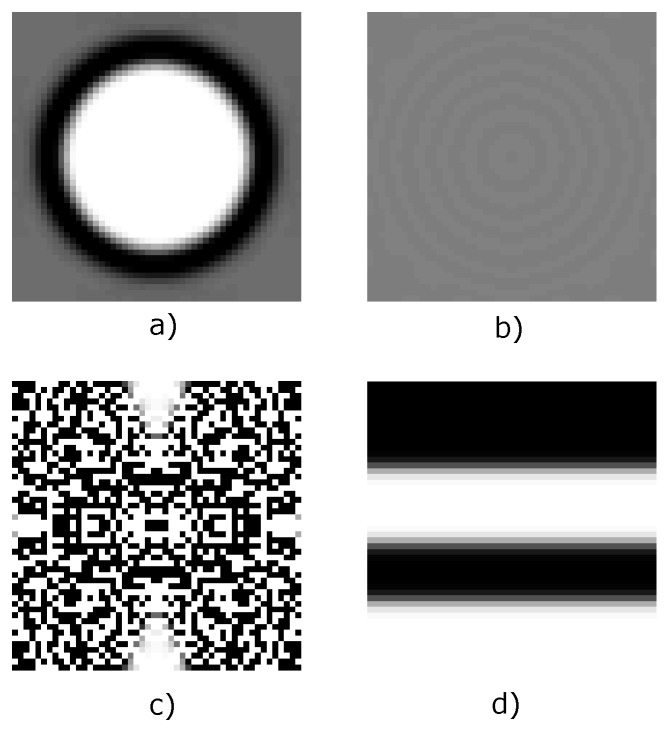
Unfeasible solutions: (**a**) partially grey. (**b**) fully grey. (**c**) early convergence (**d**) disconnected topology.

The initial guess and filter radius are not the only factors that affect the solution, in addition, the penalization power *p*, filter type, mesh size and updating scheme have also an important role in the solution. A fast and robust algorithm to solve topology optimization problems should be able to reach a feasible solution in a wide range of values for all the optimization parameters. The solution is considered feasible if:

- It is not a Grey solution (Figure 11a,b show examples of gray solutions, which are not desirable)
- The convergence is reached within a reasonable number of iterations (500 it was considered for all test)
- The final topology is not disconnected (see Figure 11d)
- Early convergence is not presented (see Figure 11c)

**Figure 12:**
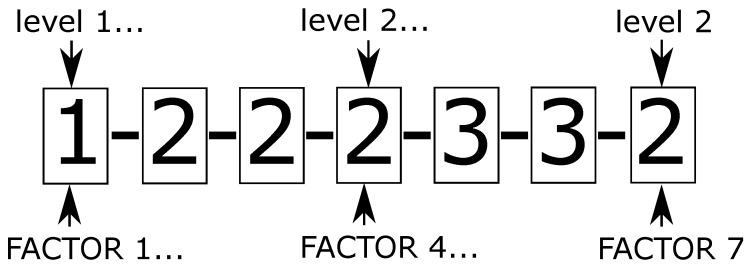
Coding of runs. The number enclosed in the rectangle represent the level and the number location represent the factor.

To analyze the interaction of all aforementioned optimization parameters, the runs analogous to a full factorial design of experiments [41] were completed, considering the factors and levels presented in the Table 1. Taking into account all the levels combinations a total of 1478 runs are obtained. Once completed all the runs, were found only 980/1478 feasible solutions, according to the feasibility criteria defined above. The obtained results are summarized in the Table 2, with the best optimal solution ID (as defined in Figure 12) for each level, and the number of feasible solutions.The most robust solver found in the analysis was the MMA, with this method, were obtained a large mount of feasible solutions when it was used in combination with all the other parameters, the best optimum value obtained with this method was achieved using the heaviside filter, a filtering radius $r_{min}=3.0$, penalization factor p=5, a mesh of 150 × 150, an uniform grid initial guess, and the filter applied to the sensitivity field.

In order to obtain a detailed view of each solver’s performance, the same results presented in Table 2 are shown in Table 3, Table 4 and Table 5 but displaying the results of each solver in combination with all the other levels.

In the Table 3 can be observed that all the early convergence solutions were obtained using the optimality criteria method, with the fully random initial guess, in fact, feasible solutions are only obtained using the optimality criteria with the center hole initial guess which is relatively close to the global optima. The MMA method proved to be the most robust, achieving convergent solutions starting from all the initial guesses, the method fails when the filter is applied to the density field instead of the sensitivity field. On the other hand, the GCMMA had problems to reach convergent solutions starting from an uniform grid initial guess, with small penalization factors and with the Heaviside filter scheme. An advantage of GCMMA is that convergent solutions can be obtained applying the filter to the density, as well as, the sensitivity field. A generalized difficulty, to get feasible solutions using the heaviside projection filter was observed. The codes implemented to obtain the results presented in this work, can be found in the Supplementary Materials.

## 9. Conclusions

The influence of optimization parameters in the solution of inverse homogenization problems using the SIMP method was studied, in addition, the features of the objective function were analyzed and it was demonstrated numerically that for some solvers and filter schemes the optimization problem become ill posed. The MMA was found as the most robust solver. Using the field average filter were obtained the largest amount of feasible solutions, and the heaviside projection filter presented problems to achieve convergent solutions in combination with the most of the studied parameters. The choice of the initial guess demonstrated to be a determining factor in the final topologies obtained. 

## Figures and Tables

**Table 1:** Factors and levels definition for all runs.

Factor ID	Factor	Levels	Level ID
1	$r_{min}$	3.0	1
		4.0	2
		5.0	3
2	Optimization solver	OC	1
		MMA	2
		GCMMA	3
3	Penalization factor	3.0	1
		5.0	2
		7.0	3
4	Filter type	Dens Average	1
		Heaviside projection	2
		Helmholtz PDE	3
5	Initial guess	Center Hole	1
		Fully random	2
		Uniform grid	3
6	Mesh size	50×50	1
		100×100	2
		150×150	3
7	Filter field	Density	1
		Sensitivity	2

**Table 2:** Solutions obtained considering all levels combination. The next acronyms are adopted: N.F.S = number of feasible solutions, N.E.C = number of early convergence solutions, N.D.G = number of disconnected geometry solutions, B.O = best optima according the coding system of Figure 12.

Factor	Levels	N.F.S	N.E.C	N.D.G	B.O	Bulk Modulus
$r_{min}$	3.0	57	2	2	1-2-2-2-3-3-2	0.1851
	4.0	48	9	1	2-3-3-3-2-3-1	0.1696
	5.0	35	8	3	3-1-2-1-1-3-2	0.1672
Updating scheme	OC	26	19	0	1-1-3-3-1-3-2	0.1713
	MMA	68	0	6	1-2-2-2-3-3-2	0.1851
	GCMMA	46	0	0	1-3-3-1-1-3-2	0.1707
Penalization factor	3.0	5	7	2	2-1-1-1-1-2-2	0.1676
	5.0	63	5	2	1-2-2-2-3-3-2	0.1851
	7.0	72	7	2	1-2-3-2-3-3-2	0.1850
Filter type	Dens Average	74	5	0	1-2-3-1-3-3-2	0.1707
	Heaviside	6	0	1	1-2-2-2-3-3-2	0.1851
	Helmholtz PDE	60	14	5	1-1-3-3-1-3-2	0.1713
Initial guess	Center Hole	88	0	0	1-2-3-2-1-3-2	0.1820
	Fully random	29	19	1	2-3-3-3-2-3-1	0.1696
	Uniform grid	23	0	5	1-2-2-2-3-3-2	0.1851
Mesh size	50×50	31	13	5	3-2-1-3-3-1-2	0.1578
	100×100	58	6	0	1-3-2-1-1-2-2	0.1682
	150×150	51	0	1	1-2-2-2-3-3-2	0.1851
Filter field	Density	19	19	0	2-3-3-3-2-3-1	0.1696
	Sensitivity	121	0	6	1-2-2-2-3-3-2	0.1851

**Table 3:** Solutions obtained using the optimality criteria solver. The following acronyms are adopted: N.F.S = number of feasible solutions, N.E.C = number of early convergence solutions, N.D.G = number of disconnected geometry solutions, B.S = best solution according the coding system of Figure 12.

	Optimality Criteria					
Factor	Levels	N.F.S	N.E.C	N.D.G	B.S	Bulk Modulus
$r_{min}$	3.0	12	2	0	1-1-3-3-1-3-2	0.1713
	4.0	7	9	0	2-1-1-1-1-2-2	0.1676
	5.0	7	8	0	3-1-2-1-1-3-2	0.1672
Penalization factor	3.0	3	7	0	2-1-1-1-1-2-2	0.1676
	5.0	12	5	0	3-1-2-1-1-3-2	0.1672
	7.0	11	7	0	1-1-3-3-1-3-2	0.1713
Filter type	Dens Average	14	5	0	2-1-1-1-1-2-2	0.1676
	Heaviside Projection	3	0	0	1-1-3-2-1-2-2	0.1669
	Helmholtz PDE	9	14	0	1-1-3-3-1-3-2	0.1713
Initial guess	Center Hole	26	0	0	1-1-3-3-1-3-2	0.1713
	Fully random	0	19	0	-	-
	Uniform grid	0	-	-	-	-
Mesh size	50×50	8	13	0	1-1-2-1-1-1-2	0.1538
	100×100	15	6	0	2-1-1-1-1-2-2	0.1676
	150×150	3	0	0	1-1-3-3-1-3-2	0.1713
Filter field	Density	3	19	0	1-1-2-1-1-2-1	0.1539
	Sensitivity	23	0	0	1-1-3-3-1-3-2	0.1713

**Table 4:** Solutions obtained using the Method of Moving Asymptotes solver. The next acronyms are adopted: N.F.S = number of feasible solutions, N.E.C = number of early convergence solutions, N.D.G = number of disconnected geometry solutions, B.S = best solution according the coding system of Figure 12.

	Method of Moving Asymptotes					
Factor	Levels	N.F.S	N.E.C	N.D.G	B.S	Bulk Modulus
$r_{min}$	3.0	28	0	2	1-2-2-2-3-3-2	0.1851
	4.0	25	0	1	2-2-2-1-3-3-2	0.1684
	5.0	15	0	3	3-2-2-1-3-3-2	0.1648
Penalization factor	3.0	2	0	2	3-2-1-3-3-1-2	0.1578
	5.0	32	0	2	1-2-2-2-3-3-2	0.1851
	7.0	34	0	2	1-2-3-2-3-3-2	0.1850
Filter type	Dens Average	36	0	0	1-2-3-1-3-3-2	0.1707
	Heaviside Projection	3	0	1	1-2-2-2-3-3-2	0.1851
	Helmholtz PDE	29	0	5	1-2-3-3-1-3-2	0.1695
Initial guess	Center Hole	32	0	0	1-2-3-2-1-3-2	0.1820
	Fully random	14	0	1	1-2-2-3-2-3-2	0.1664
	Uniform grid	22	0	5	1-2-2-2-3-3-2	0.1851
Mesh size	50×50	16	0	5	3-2-1-3-3-1-2	0.1578
	100×100	26	0	0	1-2-2-1-1-2-2	0.1682
	150×150	26	0	1	1-2-2-2-3-3-2	0.1851
Filter field	Density	1	0	0	1-2-3-3-1-1-1	0.1414
	Sensitivity	67	0	6	1-2-2-2-3-3-2	0.1851

**Table 5:** Solutions obtained using the Global Method of Moving Asymptotes solver. The next acronyms are adopted: N.F.S = number of feasible solutions, N.E.C = number of early convergence solutions, N.D.G = number of disconnected geometry solutions, B.S = best solution according the coding system of Figure 12.

	Global Method of Moving Asymptotes					
Factor	Levels	N.F.S	N.E.C	N.D.G	B.S	Bulk Modulus
$r_{min}$	3.0	17	0	0	1-3-3-1-1-3-2	0.1707
	4.0	16	0	0	2-3-3-3-2-3-1	0.1696
	5.0	13	0	0	3-3-2-1-1-3-2	0.1655
Penalization factor	3.0	0	-	-	-	-
	5.0	19	0	0	2-3-2-1-1-3-2	0.1684
	7.0	27	0	0	1-3-3-1-1-3-2	0.1707
Filter type	Dens Average	24	0	0	1-3-3-1-1-3-2	0.1707
	Heaviside Projection	0	-	-	-	-
	Helmholtz PDE	22	0	0	2-3-3-3-2-3-1	0.1696
Initial guess	Center Hole	30	0	0	1-3-3-1-1-3-2	0.1707
	Fully random	15	0	0	2-3-3-3-2-3-1	0.1696
	Uniform grid	1	0	0	2-3-3-1-3-1-2	0.1379
Mesh size	50×50	7	0	0	1-3-2-1-1-1-2	0.1535
	100×100	17	0	0	1-3-2-1-1-2-2	0.1682
	150×150	22	0	0	1-3-3-1-1-3-2	0.1707
Filter field	Density	15	0	0	2-3-3-3-2-3-1	0.1696
	Sensitivity	31	0	0	1-3-3-1-1-3-2	0.1707

## Supplementary Materials

The following are available online at https://www.mdpi.com/1996-1944/12/22/3736/s1. The information corresponding to the replication of results is presented in the supplementary material document.
