# Supplementary material for: Influence of Density-Based Topology Optimization Parameters on the Design of Periodic Cellular Materials
Source: Materials (Basel). 2019 Nov 13;12(22):3736. doi: 10.3390/ma12223736 (PMC6888502; doi:10.3390/ma12223736)
Supplement: Supplementary file 1 [file materials-12-03736-s001.zip › materials-621912-supplementary/Supplementary_Information.pdf]

Supplementary Materials

Hugo A. Alvarez[a,*], Habib R. Zambrano[a], Olavo M. Silva[b]

[a] *Universidad del Norte. Km 5 vía Puerto Colombia*
[b] *Departamento de Engenharia Mecânica, Universidade Federal de Santa Catarina. Trindade - Florianópolis*

1. General considerations

The computational implementations used to perform the analysis presented in this work were based on the Matlab codes available in the article [1] for topology design of periodic cellular materials with extreme properties using an energy-based homogenization approach. The implementations of filter procedures were defined according to the Matlab codes written by Andreassen et al. [2] and the implementations of MMA and GCMMA optimization solvers were provided by the author Svanberg [3, 4] under request.

2. Initial guesses

Three different types of initial guesses were studied in this article:

2.1. Center hole

If `x` represents an $nelx \times nely$ matrix of design variables, and `volfrac` is the volume fraction, then, an initial guess with a central hole can be coded in Matlab as:

```
x = repmat(volfrac,nely,nelx);
 for i = 1:nelx
     for j = 1:nely
```

*Corresponding author
Email address: haalvarez@uninorte.edu.co (Hugo A. Alvarez)

```
        if sqrt((i-nelx/2-0.5)^2+(j-nely/2-0.5)^2) < min(nelx,nely)/3
            x(j,i) = volfrac/2;
        end
    end
end
```

2.2. Fully random

```
ind1 = nelx/2;
ind2 = nelx;
ind3 = 1+nelx/2;
x = zeros(nely,nelx);
InixPhysQX = volfrac*round(rand(nelx/2,nely/2));
x(1:ind1,1:ind1) = InixPhysQX;
x(ind3:ind2,1:ind1) = flipud(InixPhysQX);
x(1:ind1,ind3:ind2) = fliplr(InixPhysQX);
x(ind3:ind2,ind3:ind2) = flipud(x(1:ind1,ind3:ind2));
```

2.3. Uniform grid

Using `nelx = 100` and `nely = 100` an uniform grid initial guess with an spacing of 7 columns can be coded as:

```
x = repmat(volfrac,nely,nelx);
kk = 0;
for kk = 5:7:nelx-1
        x(:,kk) = ones(nely,1);
        x(kk,:) = ones(1,nelx);
end
```

3. Solution of the finite element problem

The Matlab implementation for a plane stress problem considering a unit cell with periodic boundary conditions was presented by Xia and Breitkopf [1] to obtain the homogenized stiffness tensor C^H_{ijkl} and its derivative $\frac{\partial C_{ijkl}(\rho)}{\partial \rho}$ with a penalization factor `penal` and a material distribution `xPhys` as:

```
function [Q, dQ] = FEA(nelx,nely,xPhys,penal)
% %% MATERIAL PROPERTIES
E0 = 1.;
Emin = 1E-9;
nu = 0.3;
%% PREPARE FINITE ELEMENT ANALYSIS
A11 = [12 3 -6 -3; 3 12 3 0; -6 3 12 -3; -3 0 -3 12];
A12 = [-6 -3 0 3; -3 -6 -3 -6; 0 -3 -6 3; 3 -6 3 -6];
B11 = [-4 3 -2 9; 3 -4 -9 4; -2 -9 -4 -3; 9 4 -3 -4];
B12 = [ 2 -3 4 -9; -3 2 9 -2; 4 9 2 3; -9 -2 3 2];
KE =E0/(1-nu^2)/24*([A11 A12;A12' A11] + E0*nu*[B11 B12;B12' B11]);
nodenrs = reshape(1:(1+nelx)*(1+nely),1+nely,1+nelx);
edofVec = reshape(2*nodenrs(1:end-1,1:end-1)+1,nelx*nely,1);
edofMat = repmat(edofVec,1,8)+repmat([0 1 2*nely + [2 3 0 1] -2 -1],nelx*nely,1);
iK = reshape(kron(edofMat,ones(8,1))',64*nelx*nely,1);
jK = reshape(kron(edofMat,ones(1,8))',64*nelx*nely,1);
%---------------------------------------------------------------------------
e0 = eye(3);
ufixed = zeros(8,3);
U = zeros(2*(nely+1)*(nelx+1),3);
alldofs = (1:2*(nely+1)*(nelx+1));
n1 = [nodenrs(end,[1,end]),nodenrs(1,[end,1])];
d1 = reshape([(2*n1-1);2*n1],1,8);
n3 = [nodenrs(2:end-1,1)',nodenrs(end,2:end-1)];
d3 = reshape([(2*n3-1);2*n3],1,2*(nelx+nely-2));
n4 = [nodenrs(2:end-1,end)',nodenrs(1,2:end-1)];
d4 = reshape([(2*n4-1);2*n4],1,2*(nelx+nely-2));
d2 = setdiff(alldofs,[d1,d3,d4]);
for j = 1:3
  ufixed(3:4,j) = [e0(1,j),e0(3,j)/2;e0(3,j)/2,e0(2,j)]*[nelx;0];
  ufixed(7:8,j) = [e0(1,j),e0(3,j)/2;e0(3,j)/2,e0(2,j)]*[0;nely];
  ufixed(5:6,j) = ufixed(3:4,j) + ufixed(7:8,j);
end
wfixed = [repmat(ufixed(3:4,:),nely-1,1);repmat(ufixed(7:8,:),nelx-1,1)];
%---------------------------------------------------------------------------
qe = cell(3,3);
```

```
Q = zeros(3,3);
dQ = cell(3,3);
%% FE-ANALYSIS
sK = reshape(KE(:)*(Emin+xPhys(:)'.^penal*(E0-Emin)),64*nelx*nely,1);
K = sparse(iK,jK,sK); K = (K + K')/2;
Kr = [K(d2,d2), K(d2,d3)+K(d2,d4);K(d3,d2)+K(d4,d2), K(d3,d3)+K(d4,d3)+K(d3,d4)+K(d4,d4)];
U(d1,:) = ufixed;
U([d2,d3],:) = Kr\(-[K(d2,d1); K(d3,d1) + K(d4,d1)]*ufixed -[K(d2,d4); K(d3,d4) + K(d4,d4)]*wfixed);
U(d4,:) = U(d3,:)+wfixed;

for i = 1:3
  for j = 1:3
        U1 = U(:,i); U2 = U(:,j);
        qe{i,j}=reshape(sum((U1(edofMat)*KE).*U2(edofMat),2),nely,nelx)/(nelx*nely);
        Q(i,j) = sum(sum((Emin+xPhys.^penal*(E0-Emin)).*qe{i,j}));
        dQ{i,j} = penal*(E0-Emin)*xPhys.^(penal-1).*qe{i,j};
  end
end
```

4. Objective function

In order to evaluate the objective function for the maximum shear, bulk modulus and the general inverse homogenization function, the homogenized stiffness tensor `Q` and its derivative `dQ` should be supplied as an input. The function to be evaluated has to be uncommented from the code below:

```
function [c, dc] = objfun(Q,dQ)
%Maximum bulk modulus
    c = -0.25*(Q(1,1)+Q(2,2)+Q(1,2)+Q(2,1));
    dc = -0.25*(dQ{1,1}+dQ{2,2}+dQ{1,2}+dQ{2,1});
%Maximum shear modulus
%     c = -Q(3,3);
%     dc = -dQ{3,3};
```

```
%General inverse homogenization
%H0 = [0.3232 0.0470 0.; 0.0470 0.3232 0.; 0. 0. 0.0276];
%H1  = [Q(1,1) Q(1,2) 0.; Q(2,1) Q(2,2) 0.;0. 0. Q(3,3)];
%
%c = ((H0(1,1) - H1(1,1))^2 + (H0(1,2) - H1(1,2))^2 + (H0(2,1) -...
%H1(2,1))^2 + (H0(2,2) - H1(2,2))^2 + (H0(3,3) - H1(3,3))^2);
%dc =   -2*((H0(1,1) - H1(1,1))*dQ{1,1} + (H0(1,2) - H1(1,2))*dQ
    {1,2} +...
%(H0(2,1) - H1(2,1))*dQ{2,1}+ ...
%(H0(2,2) - H1(2,2))*dQ{2,2} + (H0(3,3) - H1(3,3))*dQ{3,3});
```

5. Monotonicity test

The "monotonicity" study was carried out varying the design variable ρ_e in the range $0.0 \leq \rho_e \leq 1.0$ with increments of 0.05 and evaluating the objective function for each ρ_e. The nodes to be evaluated were saved on the `Nodes_Monotonicity.mat` file in order to have the same elements for all the tests. To write the `.mat` file, the lines `25 and 28` should be uncommented, and after to get the `.mat` file, these lines can be commented again. "Monotonicity" is verified using the Matlab function `ismonotonic` available in the website [5].

```
1 %Mononicity study
2 clear all; clc; close all
3 xPhys = dlmread('RandomQS100X100.txt');
4 nelx = length(xPhys(:,1));
5 nely = length(xPhys(1,:));
6 penal = 5.;
7 incr = 0.05;
8 mon = zeros(length([0:incr:1]),2);
9 loop = 0;
10 leftb = randperm(100,10);
11 rightb = randperm(100,10) + nelx*(nely-1);
12 auxtop = [1:nely:nelx*(nely-1)+1];
13 topb = auxtop(randperm(10));
14 auxbottom = [nely:nelx:nelx*nely];
15 Bottomb = auxbottom(randperm(10));
16 %------------------------------------------------------------------
```

```
auxcenter1 = [1:10]+(nelx*(nely/2-5) + nelx/2);
auxcenter2 = [1:10]+(nelx*(nely/2-4) + nelx/2);
auxcenter3 = [1:10]+(nelx*(nely/2-3) + nelx/2);
auxcenter4 = [1:10]+(nelx*(nely/2-2) + nelx/2);
auxcenter5 = [1:10]+(nelx*(nely/2-1) + nelx/2);
auxcenter6 = [1:10]+(nelx*(nely/2) + nelx/2);
auxcenter = [auxcenter1,auxcenter2,auxcenter3,auxcenter4,auxcenter5,auxcenter6];
centerb = auxcenter(randperm(60));
%nodesb = [leftb, rightb, topb, Bottomb, centerb];
nodesb = load('Nodes_Monotonicity.mat');
nodesb = nodesb.nodesb;
%save('Nodes_Monotonicity.mat', 'nodesb');
for el = 1:length(nodesb);
elt = nodesb(el);
for xx = 0:incr:1
loop = loop + 1;
xPhys(elt) = xx;
[Q, dQ] = FEA(nelx,nely,xPhys,penal);
[c, dc] = objfun(Q,dQ);
mon(loop,1) = xx;
mon(loop,2) = c;
end

Name = strcat(pwd,'\','Monotonicity','\','El',int2str(elt),'.mat');
save(Name, 'mon');

fprintf('Running Element.:%5i \n',elt);
res = ismonotonic(mon(:,2));
if res == false
Name = strcat(pwd,'\','Monotonicity','\','TestNM',int2str(elt),'.mat');
save(Name, 'mon');
fprintf('Non-Monotonic Element Found:%5i \n',elt);
end
loop = 0;
mon = zeros(length([0:incr:1]),2);
end
```

```
mplot = load('Monotonicity\E19952');
mplot = mplot.mon;
plot(mplot(:,1),-mplot(:,2))
```

6. Filters implementation

The filters implementation was performed based on the codes available in the article [2]. The density and sensitivity based filter (field average), heaviside projection and the filter based on Helmholtz PDE type equation were coded in the function `applyfilter` using the `switch/case` statement. `filid` refers to the filter type (as indicated in the code comments) and `ft` indicates if the filter is applied to the sensitivity or density field. The filter implementation using the convolution `conv2` Matlab function which is equivalent to field average filter was also presented.

```
function [dc, dv] = applyfilter(dc,dv,x,H,Hs,beta,filid ,ft)
% ft = 1 density filter
% ft = 2 sensitivity filter
switch filid
case 1 %Density and sensitivity filter
if ft == 1
dc(:) = H*(x(:).*dc(:))./Hs./max(1e-3,x(:));
elseif ft == 2
dc(:) = H*(dc(:)./Hs);
dv(:) = H*(dv(:)./Hs);
end
case 2 %Using CONV2 function
if ft == 1
dc(:) = conv2(dc.*x,H,'same')./Hs./max(1e-3,x);
elseif ft == 2
dc(:) = conv2(dc./Hs,H,'same');
dv(:) = conv2(dv./Hs,H,'same');
end
case 3 %Heavyside projection
if ft == 1
```

```
    dc(:) = H*(x(:).*dc(:))./Hs./max(1e-3,x(:));
    elseif ft == 2
    dx = beta*exp(-beta*x)+exp(-beta);
    dc(:) = H*(dc(:).*dx(:)./Hs);
    dv(:) = H*(dv(:).*dx(:)./Hs);
    end
    case 4 %Based on Helmholtz type
    if ft == 1
    dc(:) = (Hs'*(H'\(H\(Hs*(dc(:).*x(:))))))./max(1e-3,x(:));
    elseif ft == 2
    dc(:) = Hs'*(H'\(H\(Hs*dc(:))));
    dv(:) = Hs'*(H'\(H\(Hs*dv(:))));
    end
    end
```

7. Frequency analysis

The frequency analysis was completed comparing all the solutions using the `corr2` Matlab built-in function which correlates two matrices. One hundred solutions were obtained starting from 100 different random initial guesses according the procedure presented in the section 2. The results were saved into a `.mat` file and then loaded to be processed in the frequency analysis. To obtain the figures of the histogram altogether with the mesostructures some manual work is required using a vector graphics application. The code implemented is presented below:

```
clear all; clc; close all
SOL = load('MaxshearAll100.mat');
xPhysALL = SOL.xPhysALL;
N = size(xPhysALL{1,1});
index = [];
AllIndex = cell(1,1);
ci = 0.9;
ind = 0;
SolSize = N(1);
indref = [1:N(1)];
```

```
ii = 1;
c = 0;
indc = indref;

while (length(indc)>1)||(isempty(indc)==true)
  c  = c + 1;
  x = xPhysALL{ii,1};
  for i = 1:length(indc)
    y = xPhysALL{indc(i),1};
    if corr2(x,y) >= ci
      ind = ind + 1;
      index(ind) = indc(i);
    end
  end
  ind = 0;
  AllIndex{c,1} = index;
  indc = setdiff(indref,index);
  ii = indc(1);
  indref = indc;
  index = [];
end

NN = size(AllIndex);
groups = zeros(NN(1),1);
histdata = zeros(99,1);
i1 = 1;
i2 = 0;
Suma = 0;
for jj = 1:NN(1)
  i1 = i2 + 1;
  i2 = i1 + length(AllIndex{jj})-1;
 MM = size(AllIndex{jj});
  valind = AllIndex{jj};
  Suma = Suma + MM(2);
  histdata(i1:i2) = jj;
end
hist(histdata,12)
```

8. Interaction among parameters

To analyze the interaction among parameters, the runs corresponding to a factorial design of experiments were studied. The Matlab function `fullfact` was used to create the different combinations of levels. The initial guesses for all the studied meshes were previously saved into `.txt` files.

```
clear all; close all; clc
%Factor 1 = rmin --> levels: 3, 4, 5
%Factor 2 = Solver --> levels: OC = 1, MMA = 2, GCMMA = 3
%Factor 3 = Penal --> levels: 3, 5, 7
%Factor 4 = Filter --> levels Density avg = 1, Heavyside Projection
    = 2,
%PDE filter = 3
%Factor 5 = Initial Guess --> Center Hole = 1, Fully random quarter
%symmetry = 2, uniform Spaced Grid = 3.

%Factor 6 = Mesh size ---> 50X50 = 1, 100X100 = 2, 150X150 = 3
%Factor 7 = Density or sensitivity filter ---> density = 1,
    sensitivity = 2

dFF = fullfact([3 3 3 3 3 3 2]);
TNOR = length(dFF(:,1));
niter = 500;
volfrac = 0.5;
tol = 0.001;
rmin_A = [3, 4, 5];
penal_A = [3., 5., 7.];
filid_A = [1, 3, 4];
nelx_A = [50, 100, 150];
nely_A = [50, 100, 150];
IGuess = cell(3,3);
IGuess{1,1}= dlmread('CentralHole50X50.txt');
IGuess{1,2}= dlmread('CentralHole100X100.txt');
IGuess{1,3}= dlmread('CentralHole150X150.txt');
%%
IGuess{2,1} = dlmread('RandomQS50X50.txt');
IGuess{2,2} = dlmread('RandomQS100X100.txt');
IGuess{2,3} = dlmread('RandomQS150X150.txt');
```

```
%%
IGuess{3,1} = dlmread('Grid50X50.txt');
IGuess{3,2} = dlmread('Grid100X100.txt');
IGuess{3,3} = dlmread('Grid150X150.txt');
%%
for i = 1417:1417
    rmin = rmin_A(dFF(i,1));
    penal = rmin_A(dFF(i,3));
    nelx = nelx_A(dFF(i,6));
    nely = nely_A(dFF(i,6));
    filid = filid_A(dFF(i,4));
    x = IGuess{dFF(i,5),dFF(i,6)};
    ft = dFF(i,7);
    Solver = dFF(i,2);
    switch Solver
        case 1
            tic
            [xPhys, c, Conv, flag] = topX_V02(nelx,nely,x,tol,
                niter,volfrac,filid,penal,rmin,ft);
            Time = toc;
        case 2
            tic
            [xPhys, c, Conv, flag] = topXMMA_V02(nelx,nely,x,tol,
                niter,volfrac,filid,penal,rmin,ft);
            Time = toc;
        case 3
            tic
            [xPhys, c, Conv, flag] = topXGCMMA(nelx,nely,x,tol,
                niter,volfrac,filid,penal,rmin,ft);
            Time = toc;
    end
    Name = strcat(pwd,'\','solutions','\','run',int2str(i),'.mat');
    SOL.c = c;
    SOL.vc = mean(xPhys(:));
    SOL.flag = flag;
    SOL.obj = 'max_shear';
    SOL.time = Time;
    SOL.conv = Conv;
```

```
    SOL.xphys = xPhys;
    save(Name, '-struct', 'SOL');
    close all; clc
end
```

9. Optimization solvers

The implemented code for the optimality criteria method was based on [1, 2]. The codes for MMA and GCMMA were supplied under request by the author Svanberg [3, 4] and can not be included on this submission.

```
function [xPhys, c, Conv, flag] = topX_V02(nelx,nely,x,tol,niter,
    volfrac,filid,penal,rmin,ft)

%% PREPARE FILTER
[H, Hs] = filterop(nelx,nely,rmin,filid);
change = 1;
loop = 0;
loopbeta = 0;
betal = 1;
%maxiter = 3;
xPhys = x;
flag = false;
maxiter = niter;
%% START ITERATION
while (change > tol)&&(loop<niter)
    loop = loop + 1;
    loopbeta = loopbeta + 1;
    if loop >= maxiter
        flag = true;
    end
    %% FE-ANALYSIS
    [Q, dQ] = FEA(nelx,nely,xPhys,penal);
    %%OBJECTIVE FUNCTION
    [c, dc] = objfun(Q,dQ);
    dv = ones(nely,nelx);
    %%FILTERING/MODIFICATION OF SENSITIVITIES
    [dc, dv] = applyfilter(dc,dv,xPhys,H,Hs,betal,filid,ft);
```

```
    %% OPTIMALITY CRITERIA UPDATE OF DESIGN VARIABLES AND PHYSICAL DENSITIES
    l1 = 0; l2 = 1e9; move = 0.2; betal = 1;
    while (l2-l1 > 1e-9)
        lmid = 0.5*(l2+l1);
        xnew = max(0,max(x-move,min(1,min(x+move,x.*sqrt(-dc./dv/lmid)))));
        [xPhys(:)] = applyfilterxudp(xnew,H,Hs,betal,filid,ft);
        if mean(xPhys(:)) > volfrac, l1 = lmid; else l2 = lmid; end
        if filid == 3 && betal < 512 && (loopbeta >= 50 || change <= 0.01)
            betal = 2*betal;
            loopbeta = 0;
            change = 1;
        end
    end
    change = max(abs(xnew(:)-x(:)));
    x = xnew;
    %%
    Conv(loop) = c;
    %PRINT RESULTS
    fprintf('It.:%5i Obj.:%11.4f Vol.:%7.3f ch.:%7.3f \n',loop,c,mean(xPhys(:)),change);
    figure(1)
    plot(loop,c,'k.')
    hold on
    figure(2)
    %% PLOT DENSITIES
    colormap(gray); imagesc(1-xPhys); caxis([0 1]); axis equal; axis off; drawnow;
end
```

9.1. Prepare filter

An auxiliary function to prepare the filer information is presented below:

```
function [H, Hs] = filterop(nelx,nely,rmin,filid)
%filid = 1: sensitivity filter (density average)
%filid = 2: convolution filter based in conv2 function
```

```matlab
%filid = 3: Heavyside projection
%filid = 4: PDE filter

%switch filid
if (filid == 1)||(filid == 3)
    iH = ones(nelx*nely*(2*(ceil(rmin)-1)+1)^2,1);
    jH = ones(size(iH));
    sH = zeros(size(iH));
    k = 0;
    for i1 = 1:nelx
        for j1 = 1:nely
            e1 = (i1-1)*nely+j1;
            for i2 = max(i1-(ceil(rmin)-1),1):min(i1+(ceil(rmin)-1),nelx)
                for j2 = max(j1-(ceil(rmin)-1),1):min(j1+(ceil(rmin)-1),nely)
                    e2 = (i2-1)*nely+j2;
                    k = k + 1;
                    iH(k) = e1;
                    jH(k) = e2;
                    sH(k) = max(0,rmin-sqrt((i1-i2)^2+(j1-j2)^2));
                end
            end
    end
end

H = sparse(iH,jH,sH);
Hs = sum(H,2);
elseif (filid == 2)
    [dy,dx] = meshgrid(-ceil(rmin)+1:ceil(rmin)-1,-ceil(rmin)+1:ceil(rmin)-1);
    H = max(0,rmin-sqrt(dx.^2+dy.^2));
    Hs = conv2(ones(nely,nelx),H,'same');
else
    Rmin = rmin/2/sqrt(3);
    KEF = Rmin^2*[4 -1 -2 -1; -1 4 -1 -2; -2 -1 4 -1; -1 -2 -1 4]/6 + ...
    [4 2 1 2; 2 4 2 1; 1 2 4 2; 2 1 2 4]/36;
```

```
    nodenrs = reshape(1:(1+nelx)*(1+nely),1+nely,1+nelx);
    edofVecF = reshape(nodenrs(1:end-1,1:end-1),nelx*nely,1);
    edofMatF = repmat(edofVecF,1,4)+repmat([0 nely+[1:2] 1],nelx*
        nely,1);
    iKF = reshape(kron(edofMatF,ones(4,1))',16*nelx*nely,1);
    jKF = reshape(kron(edofMatF,ones(1,4))',16*nelx*nely,1);
    sKF = reshape(KEF(:)*ones(1,nelx*nely),16*nelx*nely,1);
    KF = sparse(iKF,jKF,sKF);
    LF = chol(KF,'lower');
    iTF = reshape(edofMatF,4*nelx*nely,1);
    jTF = reshape(repmat([1:nelx*nely],4,1)',4*nelx*nely,1);
    sTF = repmat(1/4,4*nelx*nely,1);
    TF = sparse(iTF,jTF,sTF);
    H = LF;
    Hs = TF;
end
```

References

[1] Xia L, Breitkopf P. Design of materials using topology optimization and energy-based homogenization approach in Matlab. Structural and Multidisciplinary Optimization 2015;52(6):1229–41.

[2] Andreassen E, Clausen A, Schevenels M, Lazarov BS, Sigmund O. Efficient topology optimization in MATLAB using 88 lines of code. Structural and Multidisciplinary Optimization 2011;43(1):1–16.

[3] Svanberg K. The method of moving asymptotes-a new method for structural optimization. International journal for numerical methods in engineering 1987;24(2):359–73.

[4] Svanberg K. A Class of Globally Convergent Optimization Methods Based on Conservative Convex Separable Approximations. SIAM Journal on Optimization 2002;12(2):555–73.

[5] Cotton R. Matlab implementation of "ismonotonic" function. 2010. URL: https://www.mathworks.com/matlabcentral/fileexchange/11637-ismonotonic; accessed: 2018-05-10.
